# Kinetically Limited Bulk Polymerization of Polymer Thin Films by Initiated Chemical Vapor Deposition

**DOI:** 10.1021/acs.macromol.3c01868

**Published:** 2023-12-05

**Authors:** Varun S. Prasath, Kenneth K. S. Lau

**Affiliations:** † Department of Chemical Engineering, Rowan University, 201 Mullica Hill Road, Glassboro, New Jersey 08028, United States; ‡ Department of Chemical and Biological Engineering, Drexel University, 3141 Chestnut Street, Philadelphia, Pennsylvania 19104, United States

## Abstract

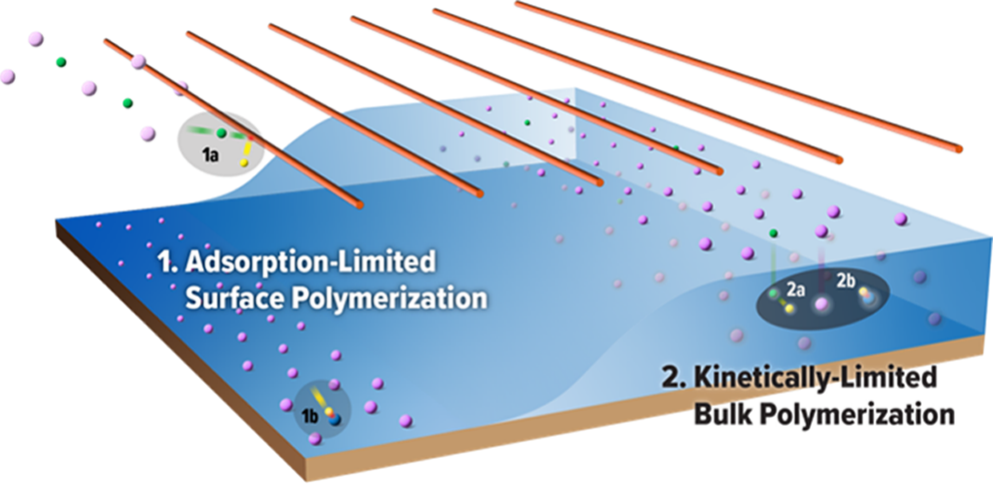

An experimental study and kinetic model analysis of the initiated chemical vapor deposition (iCVD) of polymer thin films have been performed at saturated monomer vapor conditions. Previous iCVD kinetic studies have focused on subsaturated monomer conditions where polymer deposition kinetics is known to be limited by monomer adsorption. However, iCVD kinetics at saturated conditions have so far not been systematically investigated, and it remains unclear whether the adsorption-limited phenomenon would still apply at saturation, given the abundance of monomer for reaction. To probe this question, a series of depositions of poly(vinylpyrrolidone) (PVP) thin films as a model system were performed by iCVD at substrate temperatures from 10 to 25 °C at both fully saturated (100%) and subsaturated (50%) conditions. While the deposition rates at subsaturated conditions exhibit the expected adsorption-limited behavior, the deposition rates at saturated conditions unexpectedly show two distinct deposition regimes with reaction time: an initial adsorption-limited regime followed by a kinetically limited steady-state regime. In the steady-state regime, the deposition kinetics is found to be thermally activated by raising substrate temperature with an overall activation energy of +86 kJ/mol, which agrees reasonably well with the experimentally determined value of +89 kJ/mol in the literature for bulk PVP polymerization and a mechanistically derived value of +91 kJ/mol based on the bulk free radical polymerization mechanism of PVP. These findings open new operating windows for iCVD polymerization and thin-film growth in which fast polymer deposition can be achieved without substrate cooling that can greatly simplify the iCVD scale-up to roll-to-roll processing and enable iCVD polymerization of highly volatile monomers relevant for diverse applications in biomedicine, smart wearables, and renewable energy.

## Introduction

Polymer thin films are commonly applied to fabricate devices or enhance the performance of a product. Recently, thin-film polymers have been employed in novel ways to fabricate organic light-emitting diodes,^1^ improve the power conversion efficiency of organic solar cells,^2,3^ and enhance the capacity and retention of lithium ion batteries.^4^ To fabricate thin-film polymers, a common method is to dissolve the bulk polymer into a liquid solvent and then apply the polymer solution onto the surface, for example, by dip-casting or spin-coating. The solvent is subsequently evaporated, and the dried polymer may be heated further to promote annealing and film forming.^5,6^ However, solvent-based methods suffer from drawbacks such as uneven film coating, solvent incompatibility with the underlying substrate, difficulties in coating intricate three-dimensional substrates, solvent toxicity, and residual solvent that negatively impacts polymer properties. In contrast, solvent-free methods, which in particular initiated chemical vapor deposition (iCVD), have been demonstrated to deposit thin polymer films while sidestepping the drawbacks of conventional solvent-based techniques.^7−9^ The lack of a solvent allows pristine polymer films free of solvent residue to form, mitigates material damage from liquid surface tension forces, and enables monomer functional groups to be preserved, leading to more robust polymer properties.^10−13^ The solvent-free process also leads to the deposition of highly conformal (matching surface topology) and uniform (even thickness throughout) polymer thin films onto both planar and nonplanar surfaces. As a result, iCVD has been successful in enabling a broad range of polymer-based applications and devices, including enhancing the performance of solar cells,^14^ achieving large-area stretchable electronics,^15^ creating superliquid repellent surfaces,^16^ fabricating skin-based wearable health monitors,^17^ and developing antiviral-*co*-antibacterial coatings.^18^

In iCVD, monomer and initiator vapors flow into a vacuum chamber fitted with heated filaments and a substrate that is maintained at a lower temperature than that of the rest of the reactor. The lower substrate temperature promotes monomer vapor adsorption onto the surface, while the heated filaments thermally activate the initiator in the gas phase to generate reactive species (e.g., free radicals), which then initiate surface polymerization of the adsorbed monomer.^19−21^ The amount of adsorbed monomer can be quantified by its saturation ratio, *P*_m_/*P*_sat_, which is defined as the ratio of the monomer partial pressure in the gas phase to the monomer vapor pressure evaluated at the temperature of the cooled substrate. The saturation ratio, which reflects the monomer concentration on the substrate surface, in turn controls the polymer film deposition rate under normal operating conditions.^19−21^ Under normal iCVD conditions in which the monomer at the surface is subsaturated (*P*_m_/*P*_sat_ < 1), the rate-limiting step for iCVD polymerization has been shown to be the adsorption of the monomer onto the substrate surface.^20−25^ Therefore, achieving high polymer deposition rates in iCVD typically involves lowering the substrate temperature to enhance monomer adsorption and increase *P*_m_/*P*_sat_ (by reducing *P*_sat_).^20,21^ However, the need to cool the substrate can be an operational challenge for scale-up to roll-to-roll continuous iCVD reactors, where maintaining adequate surface contact of a moving substrate against a chilled platen is difficult.^26^ Furthermore, highly volatile monomers such as ethylene oxide may require cryogenic cooling to achieve reasonably low vapor pressures (*P*_sat_ = 0.1 Torr at −32 °C^27^). The ability to achieve high deposition rates at temperatures closer to room temperature in iCVD would greatly simplify the scale-up to roll-to-roll operations, lower operating costs, and allow highly volatile monomers to be polymerized in iCVD more easily.

The aim of this study is to determine whether higher deposition rates can be achieved in iCVD at higher (rather than lower) substrate temperatures to alleviate or eliminate the requirement of substrate cooling. This would require iCVD to operate in a regime where intrinsic surface polymerization kinetics, rather than monomer vapor adsorption, controls the overall deposition rate. While previous iCVD studies have shown that the polymer deposition rate is adsorption-limited, these studies were conducted at saturation ratios well below unity.^20−25^ To date, the behavior of iCVD kinetics at saturated conditions (*P*_m_/*P*_sat_ = 1) has not been systematically scrutinized. There are a few iCVD reports that measured deposition rates at or above saturation, but no detailed studies on reaction kinetics and mechanisms were made, and these reports assumed that the kinetics still follow adsorption-limited behavior.^28,29^ Instead, we predict that the kinetic behavior will be different at saturated conditions, where adsorption is expected to be sufficiently fast to overcome adsorption limitations. We therefore hypothesize at saturated monomer conditions that the deposition rate is limited by the rate of intrinsic polymerization kinetics. To test this hypothesis and understand the mechanistic behavior at saturation, we chose the polymerization chemistry of the vinylpyrrolidone (VP) monomer to form poly(vinylpyrrolidone) (PVP) as a model system. PVP is an important material widely used in medicine,^30,31^ pharmaceutics,^32^ and consumer products.^33,34^ In addition, PVP has been successfully synthesized via iCVD^23,35^ and applied in a range of uses, for example, as a polymer electrolyte in dye-sensitized solar cells^36^ and as an antifouling coating in membrane desalination.^37^ In forming polymer coatings like PVP, the ability to find an easily scalable iCVD process that is thermally activated without substrate cooling can further broaden polymer processability and applications.

## Methods

### iCVD Process

**Figure 1:**
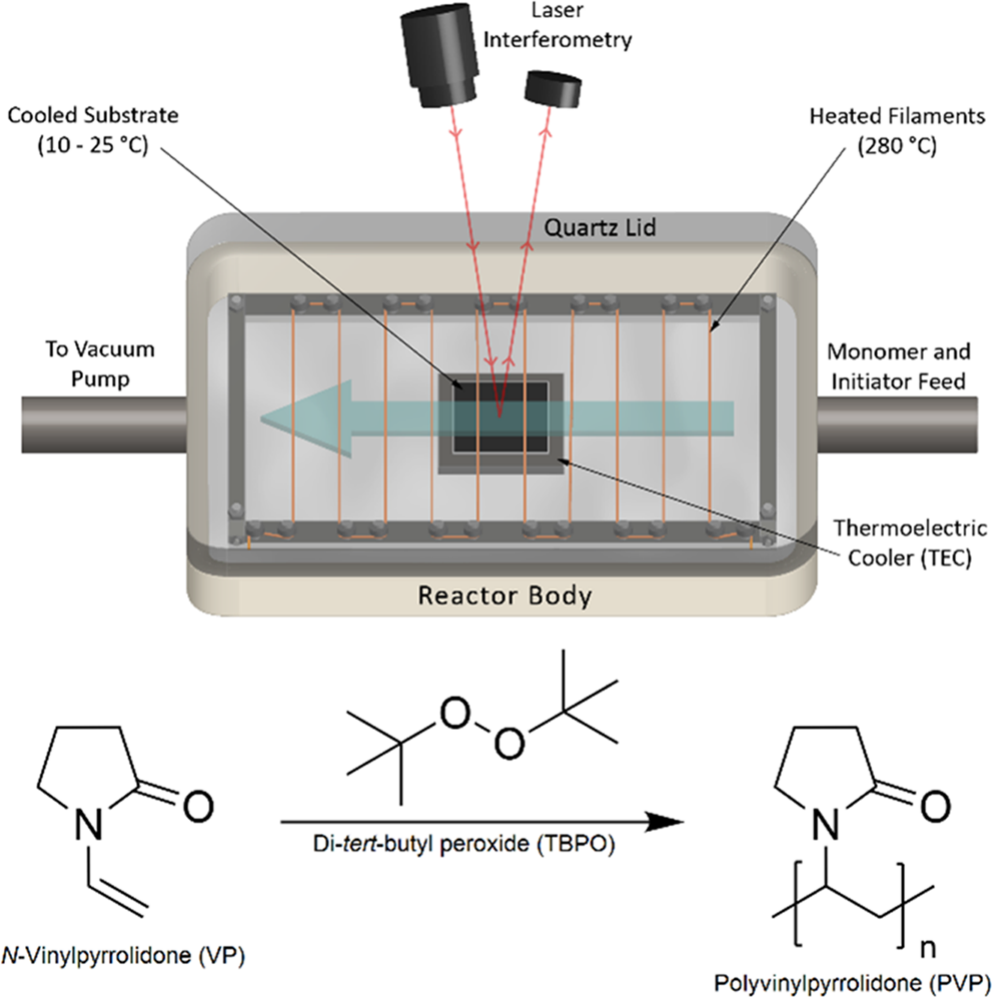
iCVD reactor scheme showing the laser interferometry setup to measure polymer film thickness vs time in situ and the polymerization reaction of the VP monomer to form a PVP polymer using TBPO as a free radical initiator.

The iCVD reactor setup and the PVP polymerization chemistry are shown in Figure 1. The monomer 1-vinyl-2-pyrrolidone (VP; 99% Millipore Sigma) and the initiator di-*tert*-butyl peroxide (TBPO; 99% Millipore Sigma), were used without further purification. The polymer, poly(vinylpyrrolidone) (PVP), was deposited onto silicon wafer substrates (University Wafer) inside a 21 × 21 × 4 cm^3^ custom-built stainless steel vacuum chamber, where the top of the reactor was enclosed with a 2.5 cm thick quartz glass window that allowed real-time tracking of polymer film growth by laser interferometry. The monomer and initiator vapors were individually metered into the reactor chamber by using precision stainless steel needle valves (Swagelok). To maintain adequate vapor flow rates, the monomer was heated to 90 °C in a glass source jar to achieve sufficient vapor pressure, while the initiator was maintained at room temperature, as its vapor pressure was sufficiently high. To thermally activate the initiator, a custom-built filament array, consisting of 12 Chromaloy O filament wires (0.5 mm diameter, GoodFellow) spaced 1.5 cm apart and placed 1.5 cm above the substrate, was resistively heated by an external DC power supply (Volteq) set at 21.4 V and 1.1 A to achieve and maintain a filament temperature of 280 °C. The substrate was cooled by a 20 cm^2^ thermoelectric cooler (Custom Thermoelectric), and by powering the thermoelectric cooler with an external DC power supply (Hewlett-Packard) through a temperature controller (Omega Engineering), the substrate temperature was maintained at a specified set point. To remove heat from the thermoelectric cooler, the cooler was placed on the reactor stage, the temperature of which was maintained by backside thermal contact with a recirculating fluid through a heater/chiller (Thermo Scientific), while the rest of the reactor was kept warm via electrical heating tape to inhibit monomer adsorption on areas outside of the substrate. The stage, substrate, and filament temperatures were measured by using K-type thermocouples (Omega Engineering). The reactor pressure was maintained by a pressure controller (MKS Instruments) connected to a pressure gauge (MKS Instruments) and a downstream butterfly valve (MKS Instruments) located between the reactor and a two-stage rotary vane vacuum pump (Edwards).

### Polymer Deposition

For our kinetic study, two series of PVP polymer depositions were carried out, with each series probing different substrate temperatures (*T*_sub_) of 10, 15, 20, and 25 °C while maintaining a fixed monomer saturation ratio, *P*_m_/*P*_sat_ (see Table 1). One deposition series was performed at subsaturated conditions of *P*_m_/*P*_sat_ = 0.5 (50% saturation), while a second series was performed at saturated conditions of *P*_m_/*P*_sat_ = 1 (100% saturation). For all depositions, the monomer (*F*_m_) and initiator (*F*_i_) vapor flow rates were maintained at 0.75 and 0.25 sccm (standard cubic centimeters per min), respectively. Thus, to maintain a fixed saturation ratio within each series at varying substrate temperatures (which changed *P*_sat_), the total reactor pressure (*P*) was adjusted accordingly (which changed the monomer partial pressure, *P*_m_ = (*F*_m_/*F*_total_) × *P*, where *F* is the molar flow rate). To probe deposition kinetics, the deposition behavior was measured in real time via an in situ laser interferometry setup consisting of a HeNe laser source (JDS Uniphase, 633 nm) and a silicon photodiode detector (Gentec-EO PH100-SI) (see Figure 1). The polymer deposition rate was estimated by counting the number of interferometry cycles (210 nm per cycle) and dividing by the corresponding elapsed reaction time (see the Supporting Information).^38^ To achieve consistent results in our study, it was important to suppress polymer deposition outside the silicon substrate area by selectively heating or thermally insulating surfaces that were not within the active deposition zone. For the deposited PVP films, the surface morphology was probed by scanning electron microscopy (SEM) using an FEI Apreo S, while the chemical composition was elucidated by Fourier transform infrared spectroscopy using a Nicolet iS50 and the FTIR spectra were compared with that of the corresponding VP monomer.

**Table 1:** iCVD Deposition Series at Fixed *P*_m_/*P*_sat_ Conditions^a^

*P*_m_/*P*_sat_	*T*_sub_ (°C)	*P*_sat_ (mTorr)	*F*_m_ (sccm)	*F*_i_ (sccm)	*P* (mTorr)
0.5	10	37	0.75	0.25	25
0.5	15	49	0.75	0.25	33
0.5	20	73	0.75	0.25	49
0.5	25	120	0.75	0.25	80
1.0	10	37	0.75	0.25	49
1.0	15	49	0.75	0.25	65
1.0	20	73	0.75	0.25	97
1.0	25	120	0.75	0.25	160

a *P*_m_ = monomer partial pressure, *P*_sat_ = monomer vapor pressure at the substrate temperature, *T*_sub_ = substrate temperature, *F*_m_ = monomer flow rate, *F*_i_ = initiator flow rate, and *P* = reactor pressure.

## Results and Discussion

### Vapor Pressure Validation

**Figure 2:**
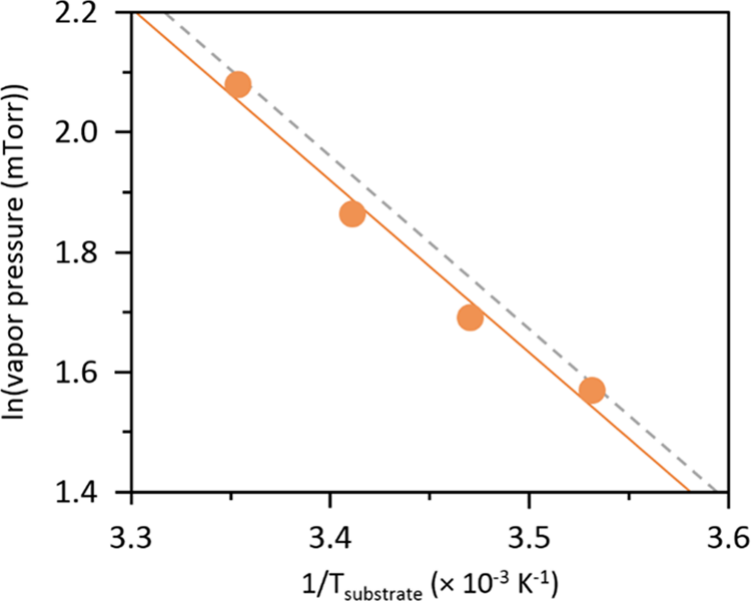
Vapor pressure data of the VP monomer (orange filled circles), plotted based on the linearized form of the Clausius–Clapeyron equation (orange line; *R*^2^ = 0.98) and compared with the fit to literature data (dotted line).

Prior to performing the PVP depositions specified in Table 1, it was important to experimentally determine the vapor pressure (*P*_sat_) of the VP monomer as a function of substrate temperature (*T*_sub_). Since the depositions hinge on keeping the saturation ratio (*P*_m_/*P*_sat_) constant at different *T*_sub_ values, accurate *P*_sat_ measurements are needed, which we can further validate with monomer vapor pressure data in the literature. We measured VP vapor pressure at a set substrate temperature (10, 15, 20, and 25 °C) using the iCVD reactor setup by flowing only VP into the reactor without initiating polymerization (no initiator flow or filament heating) and slowly ramping up the pressure until the laser interferometry signal starts to register a change, indicating the onset of monomer vapor saturation and condensation. Our measured data (solid circles) are plotted in Figure 2 and fitted to the linearized form of the Clausius–Clapeyron equation (solid line) that relates vapor pressure to temperature, *P*_sat_ = *A* exp[−Δ*H*_vap_/(*RT*)], where *A* is a pre-exponential constant, Δ*H*_vap_ is the molar vaporization enthalpy, *R* is the gas constant, and *T* is the temperature, which in our case is the substrate temperature, *T*_sub_. Based on the slope of our fitted data, the enthalpy of vaporization of the VP monomer is estimated to be 23.9 kJ/mol, which is in good agreement with the value of 24.1 kJ/mol based on fitting literature vapor pressure data to the Clausius–Clapeyron relation (see Figure 2, dotted line).^39^ The vapor pressure validation allows us to determine with confidence the reactor pressures needed to fix *P*_m_/*P*_sat_ at different substrate temperatures for the two deposition series probed (see Table 1).

### Deposition Kinetics

**Figure 3:**
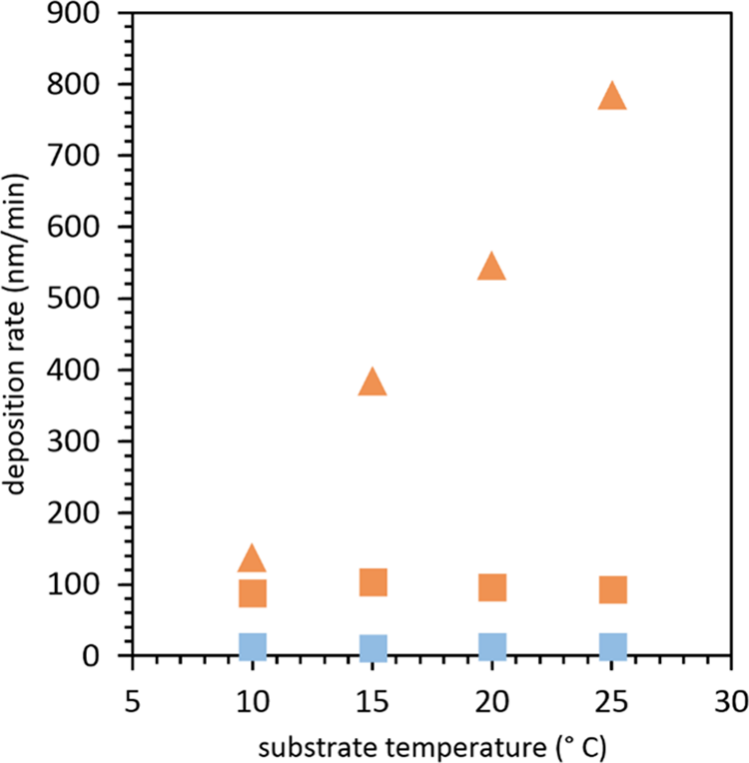
Deposition rate of the PVP polymer at different substrate temperatures for subsaturated monomer conditions (*P*_m_/*P*_sat_ = 0.5; blue) and saturated monomer conditions (*P*_m_/*P*_sat_ = 1; orange). The deposition rates at *P*_m_/*P*_sat_ = 1 are further delineated into an initial regime (orange square), followed by a later steady-state regime (orange triangle) over the course of each deposition.

To analyze the rate behavior of iCVD depositions at subsaturated and saturated conditions, two separate series of reactions, one at *P*_m_/*P*_sat_ = 0.5 and another at *P*_m_/*P*_sat_ = 1, were each performed at substrate temperatures of 10, 15, 20, and 25 °C (Table 1). For subsaturated conditions at *P*_m_/*P*_sat_ = 0.5, the deposition rate is found to be nearly constant at 14 nm/min across all substrate temperatures (see Figure 3). The deposition rate is based off of the raw laser interferometry data tracing film growth with time that shows similar cycle periods at all substrate temperatures (see the Supporting Information). This phenomenon is consistent with prior studies of iCVD deposition kinetics. At conditions below saturation, adsorption has been shown to be the rate-limiting step for polymer deposition.^20−25^ With the saturation ratio fixed at 0.5, the monomer concentration at the substrate surface is kept constant across all of the temperature runs, thereby maintaining a constant deposition rate with substrate temperature. For example, Ozaydin-Ince et al., in the iCVD deposition of poly(ethylene glycol diacrylate) (PEGDA) at a constant *P*_m_/*P*_sat_ of 0.35, found a constant deposition rate even when substrate temperature was varied.^40,41^ They attribute the constant growth rate at a fixed monomer saturation ratio to monomer adsorption (and not thermally activated surface polymerization kinetics) controlling the overall reaction rate.

While the expected deposition behavior is observed at subsaturated conditions, a different deposition behavior is seen at saturated conditions. As shown in the Supporting Information, the raw laser interferometry data at *P*_m_/*P*_sat_ = 1 for all four substrate temperature runs show two distinct deposition regimes at saturation. There is an initial regime, which is roughly the first interferometry cycle where the cycle period is longest, indicating slow deposition, that is followed by a later steady-state regime where the cycle period is much shorter, implying much faster deposition. This deposition in the steady-state regime occurs at a constant rate with time (see the Supporting Information). For these two deposition regimes at saturation, their respective deposition rates at different substrate temperatures are delineated and plotted in Figure 3. We find that the initial regime at saturation shows a constant deposition rate with the substrate temperature, which is analogous to the deposition behavior at *P*_m_/*P*_sat_ = 0.5. This suggests that the initial period of reaction at saturated conditions, like in subsaturated conditions, is governed by adsorption-limited polymerization, albeit at a faster growth rate due to the higher amount of the adsorbed monomer.

In contrast, we discover that the latter steady-state regime of a reaction at saturation shows a new deposition phenomenon that deviates from typical iCVD behavior. The deposition rate no longer remains constant but instead increases with substrate temperature. This temperature-dependent increase suggests that iCVD is no longer limited by monomer adsorption but instead is likely limited by intrinsic kinetics. Furthermore, the dramatic increase in deposition rate suggests that iCVD can reach deposition rates many fold higher at saturated conditions than is possible at subsaturated conditions, particularly by raising substrate temperature. For example, by increasing the substrate temperature from 10 to 25 °C, the deposition rate increases approximately 6-fold from 139 to 787 nm/min, which is 56-fold higher than that at 50% saturation. This new finding is in stark contrast to current iCVD understanding, where high deposition rates are thought to be achievable only with colder substrate temperatures (if the saturation ratio were allowed to vary) due to the enhanced rate of monomer adsorption.

It should be noted that the laser interferometry analysis to infer the deposition thickness and rate assumes that the laser probe area is indicative of the behavior over the entire substrate area, which requires the polymer film to be uniform across the substrate. As shown in the Supporting Information, SEM images of the deposited films show uniform morphology and minimal surface roughness that are comparable for films deposited at subsaturated and saturated conditions. In addition, the FTIR spectra of PVP films deposited at saturation, compared to the VP monomer spectrum, show that the C=C vinyl peak associated with the VP monomer disappears, which indicates that PVP polymerization is complete and no residual monomer remains in the film even under fast growth conditions at saturation (see the Supporting Information).

### Reaction Mechanisms at Saturation

**Figure 4:**
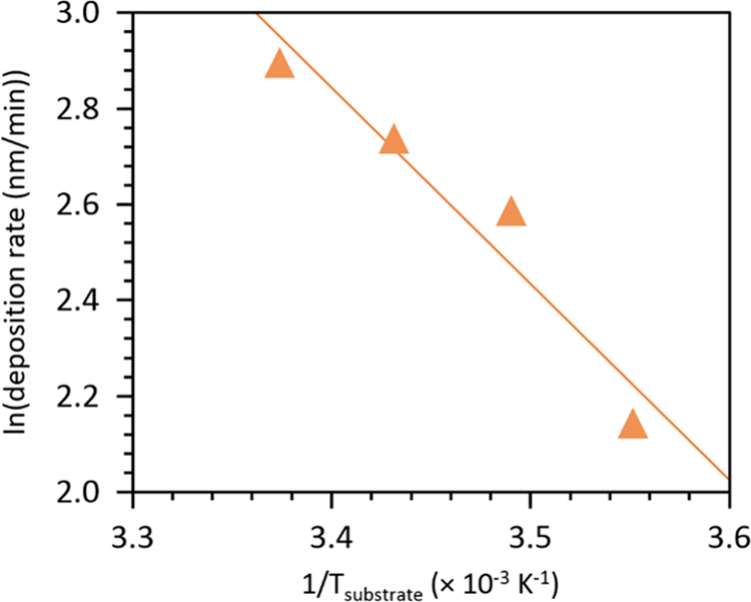
Arrhenius plot of the deposition rate data at saturated monomer conditions, *P*_m_/*P*_sat_ = 1, in the steady-state regime and the corresponding linear fit (*R*^2^ = 0.96).

To further probe the temperature-dependent trend shown in Figure 3, the deposition rates from the later steady-state regime at saturated conditions are plotted in Arrhenius form (see Figure 4). The deposition rate data fits well to an Arrhenius dependence with temperature, yielding an activation energy of +86 kJ/mol. This value is in good agreement with the reported overall activation energy of +89 kJ/mol for bulk PVP polymerization,^42^ which suggests that iCVD is kinetically limited and thermally activated during the steady-state period of polymerization. To probe this polymerization behavior mechanistically, we can also consider the elementary reactions of the free radical polymerization mechanism that drive bulk PVP polymerization. Based on the free radical mechanism (see the Supporting Information), the overall rate of polymerization can be expressed based on the limiting elementary step of polymer chain propagation:^43^
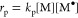
1where [M] is the monomer concentration, [M^•^] is the concentration of the propagating polymer chain radical, and *k*_p_ is the polymer propagation rate constant. By assuming the pseudosteady state of the concentrations of the polymer chain radicals [M^•^] and the initiator (primary) radicals [R^•^],
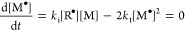
2
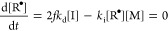
3where [*I*] is the initiator concentration, *k*_d_ is the initiator decomposition rate constant, *k*_i_ is the polymer initiation rate constant, *k*_t_ is the polymer termination rate constant, and *f* is the initiator efficiency factor (that takes into account the fraction of initiator radicals that is actually consumed by polymerization), the overall rate of polymerization can be rewritten as
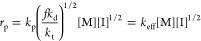
4

From the above, the temperature dependence of the effective or overall rate coefficient can be derived based on the Arrhenius temperature dependence of the individual rate coefficients and from which we can then derive the overall activation energy of bulk PVP polymerization based on the individual activation energies of the elementary steps:
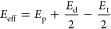
5From literature data for these individual activation energies,^44−46^ which are listed in Table 2, we can estimate the overall activation energy based on the free radical mechanism to be +91 kJ/mol. As summarized in Table 3, this mechanistic value and the experimentally measured value (+89 kJ/mol) for bulk PVP polymerization match relatively well with our iCVD PVP polymerization value (+86 kJ/mol). Such agreement strongly supports the finding that iCVD is in an intrinsic reaction-limited regime when operating under steady-state, saturated conditions.

**Table 2:** Activation Energy of Elementary Steps in the Free Radical Polymerization of PVP

elementary reaction step	activation energy (kJ/mol)	ref
initiator decomposition (*E*_d_)	151	(44)
chain propagation (*E*_p_)	20	(45)
chain termination (*E*_t_)	8	(46)

**Table 3:** Comparison of Overall Activation Energies of PVP Polymerization

	*E*_a_ (kJ/mol)	% diff.	ref
iCVD, saturated at a steady state	86		this work
bulk, experimental	89	+3.4%	(42)
bulk, free radical mechanism	91	+5.6%	(43−46)

More significantly, the close match in our overall activation energy with that of bulk PVP polymerization strongly indicates that at steady-state, saturated iCVD conditions, deposition is most likely dominated by polymerization within the bulk of the growing polymer film. This means that the entire reaction sequence of the free radical mechanism operates inside the bulk film. This is because, for the energetics of eq 5 to be valid based on the overall rate equation of eq 4, all of the elementary steps of initiator decomposition, polymer chain initiation, propagation, and termination must occur within a single common phase. For this to occur, we have to first assume that the monomer absorbs into the bulk of the growing polymer film. This assumption is reasonable due to the saturated monomer vapor conditions and the chemical affinity of the VP monomer to the corresponding PVP polymer, which would promote not only surface adsorption but also absorption into the bulk film. Even at subsaturated conditions, there is literature evidence to suggest that a monomer can absorb into the growing polymer film during iCVD deposition.^47^ Bonnet et al. found that in the iCVD deposition of neopentyl methacrylate polymer (PnPMA), enhanced deposition rates at a steady state, which they explain through a film growth model in which the bulk polymer film acts as a reservoir to hold absorbed monomer, which enhances film growth much more than what surface adsorption could provide. It is therefore conceivable that monomer absorption and bulk polymerization could become the overwhelming drivers of polymer growth, especially at saturated conditions.

For bulk polymerization to occur, besides bulk monomer absorption, we need to further assume that the initiator also absorbs into the polymer film. This deviates entirely from our understanding of the iCVD reaction mechanism at subsaturated conditions, where kinetic studies show that the initiator most likely decomposes in the gas phase to form free radicals, which then chemisorb and react with the adsorbed monomer on the surface through an Eley–Rideal mechanism.^8,41^ For our case, we have considered the possibility of an Eley–Rideal mechanism (see the Supporting Information), but such a mechanism in which the initiator does not absorb but decompose in the gas phase yields an overall activation energy that deviates from our observed experimental value. Only when we consider the bulk polymerization mechanism with initiator absorption and decomposition within the film does that lead to an overall activation energy close to our experimental one. If we are to assume initiator absorption, we have to consider the likelihood that the initiator will absorb. Although the TBPO initiator is highly volatile and therefore we might not anticipate much initiator absorption, the iCVD process is normally run where the molar ratios of the initiator to monomer vapors fed to the reactor are much higher (1:3 in our case based on their vapor flow rates) than conventional bulk or liquid phase polymerization (typically 1:100 to 1:1000). Even if we account for the difference in initiator and monomer volatilities (∼300:1), the initiator-to-monomer ratios in the absorbed phase remain comparable to that used in bulk or liquid phase polymerization. So it is therefore conceivable that a sufficient amount of the initiator could absorb to sustain the chain reaction mechanism in free radical polymerization in our case.

Finally, we have to assume that the absorption of the monomer and initiator is not adversely impacted by substrate temperature for bulk polymerization to be thermally activated through increasing the substrate temperature. For the monomer, constant saturated vapor conditions are maintained to ensure that monomer absorption is sufficiently fast and would not be rate-limiting. For the initiator, as discussed above, normal iCVD operations already demand relatively more initiator to be charged into the reactor than is typical in the liquid phase or bulk polymerization to compensate for the initiator being more volatile. This compensation is normally made in excess to the point that the initiator is generally not a limiting factor in influencing deposition kinetics.^19^ In addition, due to the free radical chain mechanism of the polymerization process, the process requires only a relatively minute amount of the initiator and resulting initiator radicals to sustain polymer chain propagation and film growth. Thus, it is reasonable to expect that there is also a sufficient initiator for our case to ensure that its absorption is efficient and would not be rate-limiting.

**Figure 5:**
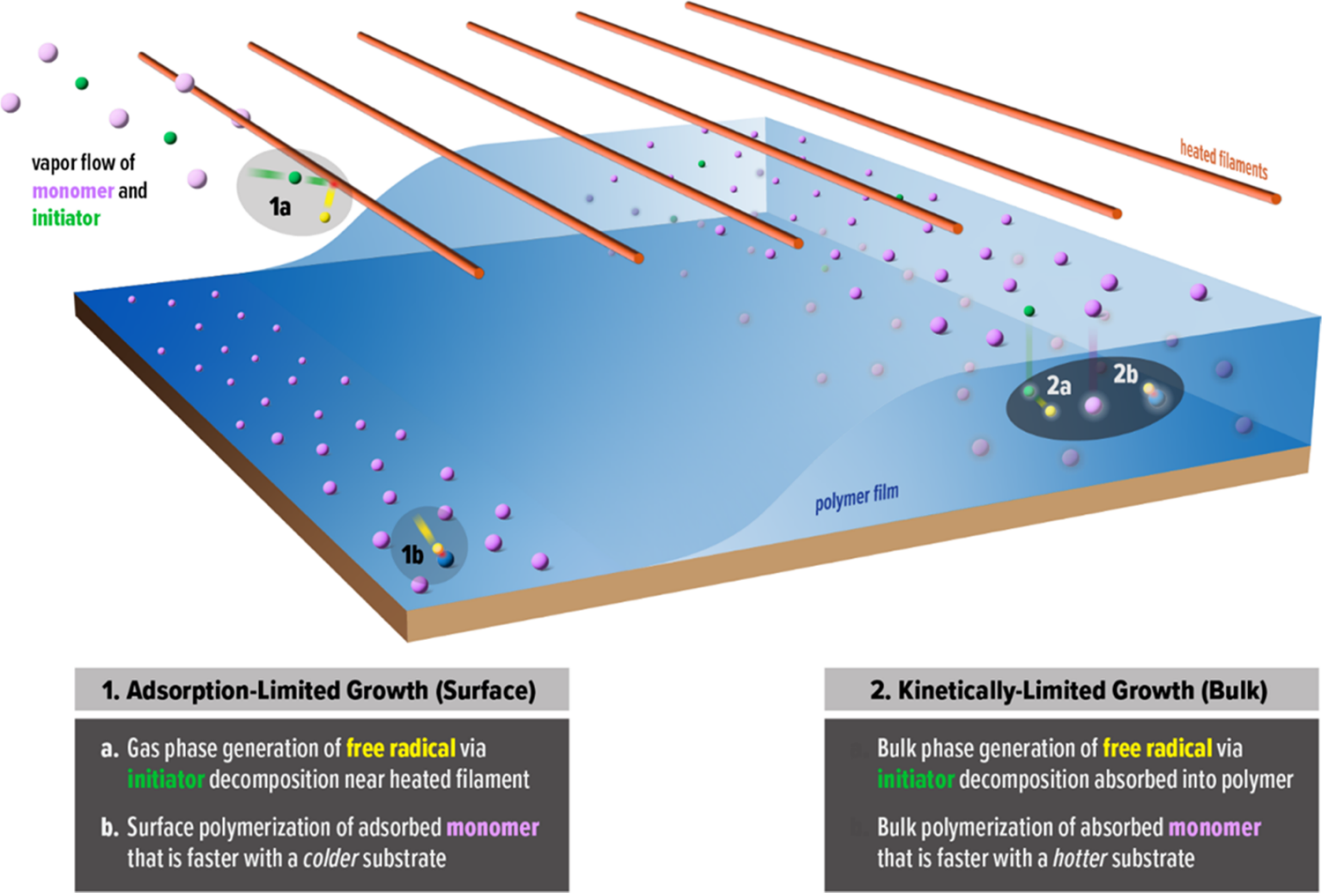
Proposed iCVD film growth model for PVP polymerization at saturated monomer conditions. (1) Initially, without a substantive polymer film on the substrate, polymer growth is confined at the surface and controlled by monomer adsorption; the initiator (green) is activated in the gas phase (1a) to form primary radicals (yellow), which initiates surface polymerization (1b) of the adsorbed monomer (purple) to form a polymer (blue film) via an Eley–Rideal mechanism. (2) At steady state, after a sufficiently thick polymer film has been established, polymer growth is dominated by bulk polymerization and governed by intrinsic bulk polymerization kinetics; the monomer and initiator absorb into the growing polymer film, leading to bulk initiator decomposition (2a) and chain growth (2b) that is thermally activated by increasing the substrate temperature.

Given all of the assumptions discussed above and their reasonable likelihood, we believe that bulk polymerization, thermally activated through substrate temperature, controls the deposition kinetics at steady-state, saturated conditions. In contrast, during the initial stage of growth at saturation, the deposition kinetics remain limited by monomer adsorption to the surface that matches the behavior at subsaturated conditions (Figure 3). To account for these two phenomena over the course of a deposition run at saturation, we propose a film growth model that is illustrated in Figure 5. At the start of the reaction, the silicon substrate is exposed and has no polymer film, so the initial polymer film growth is governed by the same adsorption-limited surface polymerization that controls the deposition at subsaturated conditions. In this initial regime, the previously established iCVD mechanism is expected to operate whereby the initiator decomposes in the gas phase and primarily initiates polymerization of the adsorbed monomer via an Eley–Rideal process at the substrate surface. As the polymer film grows, monomer and initiator molecules begin absorbing into the film, and eventually with a sufficiently thick polymer film, there is enough bulk volume to support and drive bulk polymerization that then becomes the predominant growth mechanism over surface-confined polymerization.

Our film growth model is similar to that proposed by Bonnet et al. for PnPMA deposition at subsaturated conditions.^47^ However, their growth model is for subsaturated conditions that remain entirely adsorption-limited, while our growth model is for saturated conditions in which there is, in tandem with the transition from slower initial growth to faster steady-state growth, a corresponding transition from adsorption-limited to kinetically limited growth. We should point out that there is one report of a kinetically limited iCVD deposition at subsaturated conditions.^48^ Coclite et al. have shown that for the deposition of polyhexavinyldisiloxane (PHVDSO), its deposition rate increases with substrate temperature at a fixed *P*_m_/*P*_sat_ of 0.3, and the overall activation energy matches that for the disappearance of vinyl groups consumed during polymerization. However, since there are six vinyl groups per monomer molecule that are polymerizable, we can argue for the case that the amount of reactive vinyl moieties is essentially “above the saturation” level even though the monomer molecules themselves are below saturation. In contrast, for the VP monomer, there is only one vinyl group per monomer molecule for PVP polymerization (Figure 1), so saturation of the monomer is equivalent to the saturation of the polymerizable vinyl group. So the Coclite et al. report serves to reinforce our rationale that once there is an overwhelming amount of polymerizable species available, the process becomes kinetically limited. Their report did not mention any initial adsorption-limited growth stage most likely because the deposition rates were determined based on final film thicknesses (∼200 nm) that were too thick to uncover the initial regime.

## Conclusions

An experimental study and kinetic model analysis of the iCVD of polyvinylpyrrolidone (PVP) at saturated conditions were performed. Prior iCVD kinetic studies have shown polymer deposition to be limited by monomer adsorption, but these studies were conducted primarily at subsaturated conditions. In contrast, our work has discovered a unique deposition behavior at saturated conditions. There is an initial growth regime where deposition remains adsorption-limited, similar to that at subsaturation. However, the deposition transitions to a steady-state regime, where growth becomes kinetically limited. Specifically, the deposition becomes thermally activated with the deposition rate increasing with increasing substrate temperature. The Arrhenius relationship of the steady-state deposition rate with the substrate temperature yields an overall activation energy of +86 kJ/mol. This value is in good agreement with bulk PVP polymerization of +89 kJ/mol and with a bulk free radical polymerization mechanism of +91 kJ/mol. We propose a film growth model at saturated conditions in which deposition initially occurs as an adsorption-limited surface polymerization process that then transitions to a kinetically limited bulk polymerization at a steady state, where the presence of a sufficiently thick polymer film provides a favorable medium for the monomer and initiator to absorb and enable free radical chain polymerization within the bulk film. This discovery opens a new operating window for iCVD, whereby higher deposition rates and faster polymer film processing can be achieved with higher substrate temperatures. This contrasts with previously established iCVD processing knowledge, in which higher throughput can only be achieved at colder substrate temperatures that promote monomer adsorption. We believe that the new operating window is not specific to PVP but applies more generally to the free radical polymerization of vinyl monomers. Achieving fast polymer film deposition without substrate cooling can greatly simplify the scale-up of iCVD operations, e.g., roll-to-roll systems where substrate cooling of a moving substrate is quite challenging. Furthermore, the range of polymer chemistries that can be employed in iCVD can be extended to highly volatile monomers in which a lack of adsorption at subsaturated conditions makes polymer growth impractically slow. The ability to utilize saturated monomer conditions that remove adsorption limitations and promote thermally activated processing is very attractive for iCVD processing.

## Supporting Information Available

Laser interferometry data; polymer film characterization; free radical chain polymerization mechanism in the bulk; and Eley–Rideal polymerization mechanisms (PDF)
