# Supplementary material for: Kinetically Limited Bulk Polymerization of Polymer Thin Films by Initiated Chemical Vapor Deposition
Source: Macromolecules. 2023 Dec 5;56(24):10111–8. doi: 10.1021/acs.macromol.3c01868 (PMC10753876; doi:10.1021/acs.macromol.3c01868)
Supplement: Supplementary file 1 — ma3c01868_si_001.pdf [file ma3c01868_si_001.pdf]

Supporting Information

Kinetically-Limited Bulk Polymerization of Polymer Thin Films by Initiated Chemical Vapor Deposition

Varun S. Prasath and Kenneth K. S. Lau*

1. Laser Interferometry

Laser interferometry setup. Figure S1 shows a magnified view of the laser interferometry setup used to trace the thickness of the growing polymer film in situ. A He-Ne laser source beam is directed at the substrate at an incidence angle θ_1. Upon contact with the polymer film, a portion of the laser is reflected off the vacuum-polymer interface, and the rest is refracted through the film to reflect off the polymer-silicon interface. As the polymer film grows, these reflections result in cycles of constructive (in phase) and destructive (out of phase) interference in the overall reflected laser beam, which gives rise to a sinusoidal interferometry response. Based on the properties of light interference, each sinusoidal cycle can be directly related to a cycle thickness, h, of the growing polymer film as:[1]

$$h = \frac{\lambda \cos\theta_2}{2n_2} \qquad \text{(S1)}$$

where λ is the wavelength of the laser beam (632.8 nm in our case), θ_2 is the angle of refraction, and n_2 is the index of refraction of the polymer film (~1.5). The incident angle for our experiments (θ_1) was set at 10°, while the refracted angle can be deduced using Snell's law:

$$n_1 \sin\theta_1 = n_2 \sin\theta_2 \qquad \text{(S2)}$$

where the index of refraction of the vacuum environment above the film, n_1, is equal to 1. Furthermore, we can use the Pythagorean identity to obtain an expression for $\cos\theta_2$:

$$\cos\theta_2 = \sqrt{1 - \sin^2\theta_2} \qquad \text{(S3)}$$

Combining Equations S1-S3 gives an expression relating the cycle thickness to all our known values:

$$h = \frac{\lambda}{2n_2}\sqrt{1 - \left(\frac{n_1 \sin\theta_1}{n_2}\right)^2} \qquad \text{(S4)}$$

This yields a cycle thickness of 210 nm per sinusoidal oscillation. Using this value, we can count the number

of sinusoidal cycles (N) in our laser interferometry data for each deposition run, multiply them by 210 nm/cycle (h), and divide by the reaction time (t_N) for the cycles counted to derive the film deposition rate ($= Nh/t_N$) during that period of time.

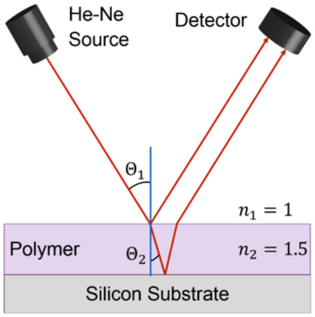

Figure S1. Laser interferometry to measure the thickness of the deposited PVP film in situ.

Laser interferometry data at sub-saturation. Figure S2 shows the raw interferometry data for P_m/P_{sat} of 0.5 at substrate temperatures of 10, 15, 20, and 25 °C. The deposition rates for all four trials ranged between 11 to 16 nm/min, which indicates that the deposition rate is relatively constant with a constant P_m/P_{sat} at sub-saturated conditions regardless of substrate temperature. This is due to the concentration of adsorbed monomer being constant with a constant P_m/P_{sat}.[2]

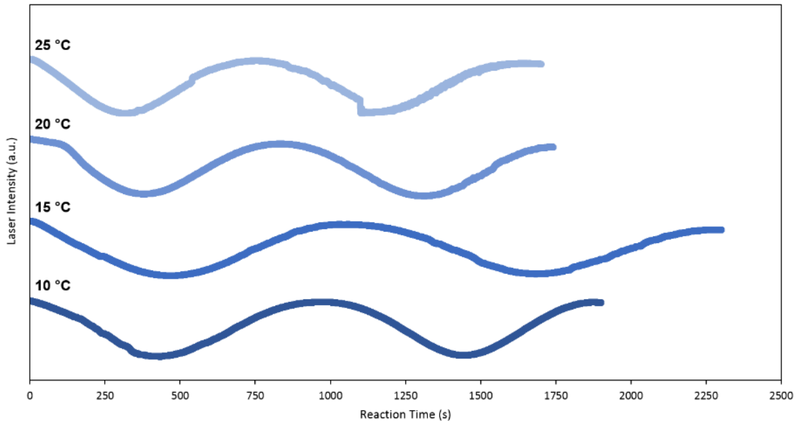

Figure S2. Laser interferometry data for P_m/P_{sat}= 0.5 at substrate temperatures of 10, 15, 20, and 25 °C (from dark to lighter shades of blue). The corresponding deposition rates at each substrate temperature are 14, 11, 15, and 16 nm/min, respectively.

Laser interferometry data at saturation. Figure S3 shows the raw interferometry data for P_m/P_{sat} of 1 at substrate temperatures of 10, 15, 20, and 25 °C. The initial transient is the first cycle (outlined by the red box) that contains a longer period than the rest of the cycles. Given that this initial period is similar for all four temperature trials (similar box width), the initial transient at saturated conditions shows a relatively constant deposition rate with substrate temperature similar to that at sub-saturated conditions (Figure S2), with rates ranging from 89 to 96 nm/min. Following the initial transient, the interferometry data shows much faster cycling in the subsequent steady state deposition regime. And unlike the initial transient, the cycling rate or deposition rate in the steady state regime increases as substrate temperature increases, rising significantly from 139 nm/min at 10 °C to 787 nm/min at 25 °C. This suggests that the steady state regime at saturated conditions is operating in a kinetically-limited, thermally-activated regime.

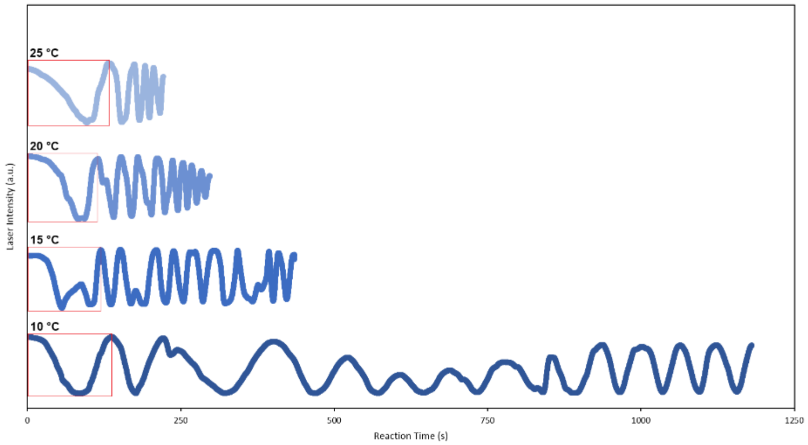

Figure S3. Laser interferometry data for P_m/P_{sat} = 1 at substrate temperatures of 10, 15, 20, and 25 °C (from dark to lighter shades of blue). The initial transient regime is indicated by the red box encompassing the first cycle of each temperature run. The steady state regime consists of all the cycles following the first. With increasing substrate temperature, the deposition rates for the initial transient are 89, 105, 99, and 96 nm/min, respectively, while that in the steady state regime are 139, 387, 547, and 787 nm/min, respectively. It should be noted that the amplitude variations of the oscillations do not affect the oscillation

period but amplitude changes could be due to instrument or process fluctuations with operating at saturated monomer conditions.

Deposition thickness vs. time. Figure S4 shows a plot of deposited PVP film thickness vs. deposition time at saturated conditions based on the raw laser interferometry data in Figure S3 in the *steady state regime* (after the first transient interferometry cycle). The origin of the plot has therefore been shifted to start *after the initial transient* i.e., at each substrate temperature, time only counts from when the steady state regime begins, and steady state film thickness only considers the additional film that grows during the steady state regime. Based on Figure S4, for each substrate temperature, the film grows fairly linearly over the course of the deposition run at steady state, and the linear increase becomes faster (steeper slope) with increasing substrate temperature.

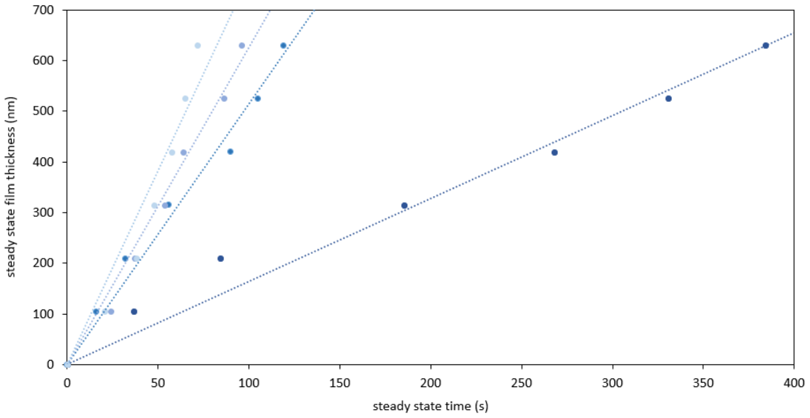

Figure S4. Plot of deposited PVP film thickness vs. deposition time, at saturation and in the steady state regime, at substrate temperatures of 10, 15, 20, and 25 °C (from dark to lighter shades of blue). In the steady state regime, the film thickness increases linearly over the course of the deposition run, and the linear rise becomes faster with increasing substrate temperature.

2. Polymer Film Characterization

Scanning electron microscopy. Figure S5 shows the SEM images of the PVP films deposited at sub-saturated and saturated monomer conditions. Qualitatively, the films appear to be uniform and there is no significant difference in physical morphology or surface roughness between the two deposition conditions.

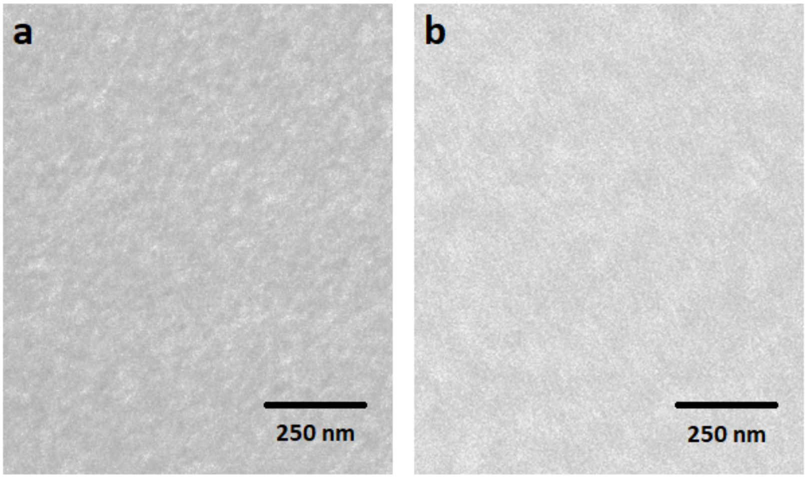

Figure S5. Top-down SEM images of the deposited PVP films at **(a)** sub-saturated, and **(b)** saturated conditions. The films from the two deposition conditions appear to be similar, showing no significant differences in physical morphology or surface roughness even with a significant increase in monomer partial pressure from sub-saturation to saturation.

Fourier transform infrared spectroscopy. Figure S6 shows the FTIR spectrum of a representative PVP film deposited at saturated monomer conditions that is compared to the FTIR spectrum of the VP monomer. The polymerization of VP to PVP leads to a loss of the vinyl C=C peak at 1620 cm^{-1}. This loss confirms the free radical polymerization across the double bond, and further indicates the lack of any residual monomer remaining in the film that might occur at saturation and with polymerization within the bulk film. For the polymer film, the spectrum further shows the expected peaks for linear PVP homopolymer, including the strong carbonyl C=O stretch at 1640 cm^{-1}, and peaks associated with C–C, C–N and CH_x stretches in the wavenumber ranges of 1400–1500, 1250–1335 and 2800–3000 cm^{-1}, respectively.[3] There

is also a broad OH stretch around 3200–3600 cm^{-1}, which is due to absorbed moisture given the strong affinity of water to PVP as a hydrogel material.

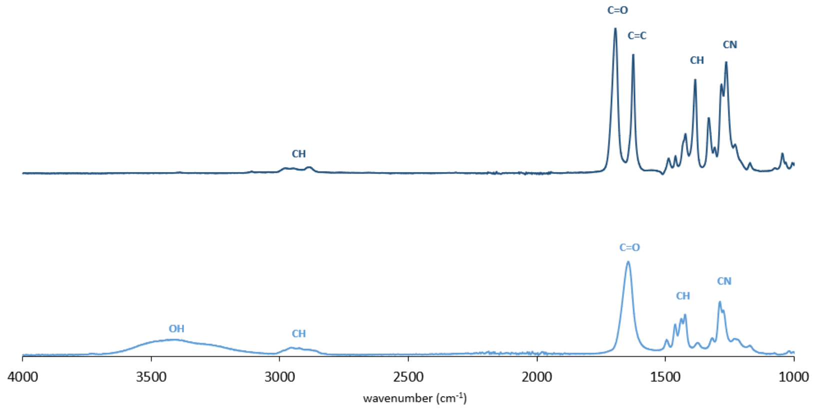

Figure S6. FTIR spectra of **(top)** *N*-vinylpyrrolidone (VP) monomer, and **(bottom)** the corresponding poly(*N*-vinylpyrrolidone) (PVP) polymer deposited at saturated conditions. The VP monomer shows the distinct vinyl C=C group at 1620 cm^{-1}, which completely disappears in the PVP polymer, indicating complete polymerization and no residual monomer remains within the bulk film even when deposited at saturation and fast kinetics.

3. Free Radical Chain Polymerization Mechanism

The mechanism for free radical chain polymerization comprises of four main elementary reactions:[4]

Initiator Decomposition	$\mathrm{I} \overset{k_\mathrm{d}}{\rightarrow} 2\mathrm{R}\cdot$	$r_\mathrm{d} = k_\mathrm{d}[\mathrm{I}]$
Initiation	$\mathrm{R}\cdot + \mathrm{M} \overset{k_\mathrm{i}}{\rightarrow} \mathrm{M}_1\cdot$	$r_\mathrm{i} = k_\mathrm{i}[\mathrm{R}\cdot][\mathrm{M}]$
Propagation	$\mathrm{M_n}\cdot + \mathrm{M} \overset{k_\mathrm{p}}{\rightarrow} \mathrm{M_{n+1}}\cdot$	$r_\mathrm{p} = k_\mathrm{p}[\mathrm{M}\cdot][\mathrm{M}]$
Termination (coupling)	$\mathrm{M_n}\cdot + \mathrm{M_m}\cdot \overset{k_\mathrm{t}}{\rightarrow} \mathrm{M_{n+m}}$	$r_\mathrm{t} = k_\mathrm{t}[\mathrm{M}\cdot]^2$
(disproportionation)	$\mathrm{M_n}\cdot + \mathrm{M_m}\cdot \overset{k_\mathrm{t}}{\rightarrow} \mathrm{M_n} + \mathrm{M_m}$	

where I is the initiator species, M is the monomer species, $\mathrm{R}\cdot$ is the initiator radical species, and $\mathrm{M}\cdot$ is the polymer chain radical species. In each respective rate law expression, r is the rate of reaction and k is the reaction rate coefficient, and where relevant the rate coefficient has been assumed to be independent of the length of the polymer chain radical. It is also generally assumed that chain propagation is the rate limiting step. For bulk polymerization, all the elementary steps occur in a single common phase and its kinetic treatment is presented in the main text.

4. Eley-Rideal Mechanism with Constant Surface Monomer Concentration

In an attempt to explain the steady state kinetics of iCVD for $P_\mathrm{m}/P_\mathrm{sat} = 1$, the Eley-Rideal reaction mechanism[5] was explored as an alternative to the bulk polymerization mechanism. Instead of both initiator radicals and monomer molecules adsorbing onto the substrate surface to undergo initiation, the Eley-Rideal mechanism proposes that only the monomer molecules strongly adsorb onto the substrate and the initiator radicals react with the monomer from the gas phase directly via molecular collisions to initiate surface polymerization. This reasoning is based on the much higher volatility of the initiator compared to the monomer in typical iCVD polymerization chemistries. Ozaydin-Ince and Gleason have found, in the iCVD depositions of ethylene glycol diacrylate (EGDA) and neopentyl methacrylate (npMA), that the initiator concentration had little effect on the deposition rate at constant $P_\mathrm{m}/P_\mathrm{sat}$ even when varying both initiator

flowrate and substrate temperature.[2] The weak dependence on the initiator concentration suggests that the initiator radicals do not adsorb onto the substrate like the monomer does, but instead could react via the Eley-Rideal mechanism of directly colliding from the gas phase with the adsorbed monomer molecules. This leads to slight but important differences compared to that of the bulk polymerization mechanism presented above in Section 2 for the first two elementary steps:

Initiator Decomposition	$\mathrm{I_g} \xrightarrow{k_\mathrm{d}} 2\mathrm{R_g}\cdot$	$r_\mathrm{d} = k_\mathrm{d}[\mathrm{I_g}]$
Initiation	$\mathrm{R_g}\cdot + \mathrm{M} \xrightarrow{k_\mathrm{i}} \mathrm{M_1}\cdot$	$r_\mathrm{i} = k_\mathrm{i}[\mathrm{R_g}\cdot][\mathrm{M}]$

where the subscript "g" specifically denotes the species (initiator and initiator radical) that remain in the gas phase in the Eley-Rideal mechanism.

Similar to the kinetic treatment presented in the main text for the bulk polymerization mechanism, the overall rate of polymerization in terms of the elementary reaction rate coefficients and species concentrations in the Eley-Rideal reaction mechanism can be derived:

$$r_\mathrm{p} = k_\mathrm{p}\sqrt{\frac{f k_\mathrm{d}}{k_\mathrm{t}}}\sqrt{[\mathrm{I_g}]}\,[\mathrm{M}] \tag{S5}$$

Now, we seek to relate the gas phase initiator concentration $[\mathrm{I_g}]$ to the monomer vapor pressure, P_sat, which in turn is related to the substrate temperature by the Clausius-Clapeyron expression. First, since $P_\mathrm{m}/P_\mathrm{sat} = 1$, it follows that:

$$P_\mathrm{m} = P_\mathrm{sat} \tag{S6}$$

The value P_m can be related to the overall reactor pressure, P, using Dalton's law of partial pressures:

$$P_\mathrm{m} = y_\mathrm{m} P \tag{S7}$$

where y_m is the fraction of monomer vapor in the reactor chamber (this value is fixed at 0.75 for all the reactions based on our chosen deposition conditions given in Table 1 in the main text). Using the ideal gas law, which is applicable for the low pressure iCVD process, an expression relating the reactor pressure to the overall vapor concentration can be obtained:

$$P = [C_g]RT_g \tag{S8}$$

where $[C_g]$ is the total vapor concentration, which is the ratio of the total moles of vapor in the reactor chamber to the total reactor volume, and T_g is the vapor temperature in the reactor chamber (not to be confused with the substrate temperature T_{sub}). Since the vapor consists mostly of monomer and initiator (here we ignore the much lower concentration of primary initiator radicals), $[C_g]$ can be directly related to the initiator vapor concentration, $[I_g]$, using the monomer vapor mole fraction, y_m:

$$[I_g] = (1 - y_m)[C_g] \tag{S9}$$

where $(1 - y_m)$ is then the initiator vapor mole fraction. Isolating $[I_g]$ and combining Equations S6–S9 above produces the following expression for $[I_g]$ as a function of monomer vapor pressure:

$$[I_g] = \frac{(1 - y_m)}{y_m}\left(\frac{P_{sat}}{RT_g}\right) \tag{S10}$$

Substituting this expression for $[I_g]$ into Equation S5 produces the following overall rate of polymerization expression:

$$R_p = k_p\sqrt{\frac{fk_d}{k_t}}\sqrt{\frac{(1 - y_m)}{y_m}\left(\frac{P_{sat}}{RT_g}\right)}\,[M] \tag{S11}$$

In Equation S11, the terms that are a function of *substrate* temperature are k_p, k_t, and P_{sat} as they relate to reactions and adsorption occurring on the substrate surface. Note that k_d is not a function of *substrate* temperature but rather the gas temperature as the initiator decomposition is assumed to occur in the vicinity of the heated filaments in the gas phase. Table S1 summarizes the expressions for k_d, k_p, k_t and P_{sat}, and their dependence on the appropriate temperature.

Although [M] is the adsorbed monomer concentration and therefore would depend on substrate temperature, we have assumed for the development of the kinetic model here that this concentration is in excess at saturation and can be considered a constant. The dependence of the rate coefficients k_p and k_t on *substrate* temperature can be expressed through their respective Arrhenius relations, and the vapor pressure, P_{sat}, can be expressed using the Clausius-Clapeyron relation (see Table S1). As a result, an overall

activation energy for the rate of polymerization can be derived:

$$E_{\mathrm{eff}} = E_{\mathrm{p}} - \frac{E_{\mathrm{t}}}{2} + \frac{\Delta H_{\mathrm{vap}}}{2} = +28\,\frac{\mathrm{kJ}}{\mathrm{mol}} \tag{S12}$$

The activation energy obtained from Equation S12 of +28 kJ/mol (based on the activation energies and heat of vaporization values given in the main text) is significantly different from our observed overall polymerization activation energy of +86 kJ/mol. Thus, the above Eley-Rideal mechanism with constant surface monomer concentration most likely does not explain our observations.

Table S1. Temperature Dependence of the Elementary Rate Constants and Vapor Pressure

Parameter Expression	*Temperature Dependence*
$k_{\mathrm{d}} = k_{\mathrm{d},0} \exp(-E_{\mathrm{d}}/RT_{\mathrm{g}})$	vapor temperature
$k_{\mathrm{p}} = k_{\mathrm{p},0} \exp(-E_{\mathrm{p}}/RT_{\mathrm{sub}})$	substrate temperature
$k_{\mathrm{t}} = k_{\mathrm{t},0} \exp(-E_{\mathrm{t}}/RT_{\mathrm{sub}})$	substrate temperature
$P_{\mathrm{sat}} = \mathrm{A} \exp(-\Delta H_{\mathrm{vap}}/RT_{\mathrm{sub}})$	substrate temperature

5. Eley-Rideal Mechanism with Equilibrated Surface Monomer Concentration

An alternative to the above approach is to consider the possibility that the surface monomer concentration is not constant, but is instead based on an equilibrium between the adsorbed monomer on the substrate surface and the monomer vapor in the reactor chamber:

$$\mathrm{M(g)} \underset{k_{\mathrm{des}}}{\overset{k_{\mathrm{ad}}}{\rightleftharpoons}} \mathrm{M(ad)} \tag{S13}$$

$$K_{\mathrm{eq}} = \frac{[\mathrm{M}]}{[\mathrm{M_g}]} = \frac{k_{\mathrm{ad}}}{k_{\mathrm{des}}} \tag{S14}$$

Based on an equilibrium between monomer adsorption and desorption in Equation S13, the equilibrium constant, K_{eq}, can be written as the ratio of the adsorbed monomer concentration on the substrate [M] to the monomer vapor concentration $[\mathrm{M_g}]$ in Equation S14, which can also be written as the ratio of the

adsorption and desorption rate coefficients, k_{ad} and k_{des}. By rearranging this expression, the adsorbed monomer concentration [M] can be expressed as:

$$[\mathrm{M}] = [\mathrm{M_g}]\frac{k_{\mathrm{ad}}}{k_{\mathrm{des}}} \tag{S15}$$

Furthermore, the concentration of monomer vapor, $[\mathrm{M_g}]$, can be related to the saturated pressure, P_{sat}, using the fact that $P_{\mathrm{m}} = P_{\mathrm{sat}}$ at saturation, and applying the ideal gas law:

$$[\mathrm{M_g}] = \frac{P_{\mathrm{m}}}{RT_{\mathrm{g}}} = \frac{P_{\mathrm{sat}}}{RT_{\mathrm{g}}} \tag{S16}$$

Equations S15 and S16 can be combined and substituted into Equation S11 to obtain an overall rate of polymerization equation in terms of the adsorption and desorption rate coefficients:

$$R_{\mathrm{p}} = k_{\mathrm{p}}\sqrt{\frac{fk_{\mathrm{d}}}{k_{\mathrm{t}}}}\sqrt{\frac{(1-y_{\mathrm{m}})}{y_{\mathrm{m}}}\left(\frac{P_{\mathrm{sat}}}{RT_{\mathrm{g}}}\right)}\left(\frac{P_{\mathrm{sat}}}{RT_{\mathrm{g}}}\right)\left(\frac{k_{\mathrm{ad}}}{k_{\mathrm{des}}}\right) \tag{S17}$$

In Equation S17, the terms that are functions of *substrate* temperature are k_{p}, k_{t}, P_{sat}, k_{ad}, and k_{des}. All other terms are constant with respect to *substrate* temperature. Based on an Arrhenius relation for each of the rate coefficients and the Clausius-Clapeyron relation for the vapor pressure, an overall activation energy for the rate of polymerization can be derived:

$$E_{\mathrm{eff}} = E_{\mathrm{p}} - \frac{E_{\mathrm{t}}}{2} + \frac{\Delta H_{\mathrm{vap}}}{2} + \Delta H_{\mathrm{vap}} + E_{\mathrm{ad}} - E_{\mathrm{des}} \tag{S18}$$

The activation energies of adsorption and desorption can be directly related to the enthalpy of desorption:

$$E_{\mathrm{des}} - E_{\mathrm{ad}} = \Delta H_{\mathrm{des}} \tag{S19}$$

The enthalpy of desorption for *N*-vinylpyrrolidone monomer is estimated to be around 20–30 kJ/mol based on adsorption and desorption measurements of similar compounds like *N*-methylpyrrolidinone.[6] Using this value and the values of the other parameters stated in the main text, the overall activation energy for this proposed mechanism can be estimated:

$$E_{\mathrm{eff}} = E_{\mathrm{p}} - \frac{E_{\mathrm{t}}}{2} + \frac{3\Delta H_{\mathrm{vap}}}{2} - \Delta \mathrm{H}_{\mathrm{des}} = 22\text{–}32\,\frac{\mathrm{kJ}}{\mathrm{mol}} \tag{S20}$$

The activation energy obtained from Equation S20 of +22 to 32 kJ/mol is far off from our observed value of +86 kJ/mol. Thus, the above Eley-Rideal mechanism with equilibrated surface monomer concentration also most likely does not explain our observations.

References

(1) Kim, S. H.; Vurens, G.; Somorjai, G. A. Direct Measurement of Polymer Growth Rate in a Model Ziegler–Natta Polymerization System Using Laser Reflection Interferometry. *J. Catal.* **2000**, *193*, 171-175.

(2) Ozaydin-Ince, G.; Gleason, K. K. Tunable Conformality of Polymer Coatings on High Aspect Ratio Features. *Chem. Vap. Deposition* **2010**, *16*, 100-105.

(3) Smolin, Y. Y.; Janakiraman, S.; Soroush, M.; Lau, K. K. S. Experimental and Theoretical Investigation of Dye Sensitized Solar Cells Integrated with Crosslinked Poly(vinylpyrrolidone) Polymer Electrolyte Using Initiated Chemical Vapor Deposition. *Thin Solid Films* **2017**, *635*, 9-16.

(4) Odian, G. Radical Chain Polymerization. In *Principles of Polymerization*, John Wiley & Sons, Inc, **2004**; pp 198-349.

(5) Eley, D. D.; Rideal, E. K. Parahydrogen Conversion on Tungsten. *Nature* **1940**, *146* (3699), 401-402.

(6) Edge, M.; Turner, D.; Liauw, C. M.; Robinson, J.; Allen, N. S. The Retention of Heterocyclics by Siliceous Frameworks. Part I: The Role of the Heterocylic. *J. Mater. Sci.* **2001**, *36*, 1443-1450.
